# Does Covera-19 know ‘when to hold ‘em or ‘when to fold ‘em? A translational thought experiment

**DOI:** 10.1186/s41231-021-00090-5

**Published:** 2021-06-30

**Authors:** Gerald Dieter Griffin

**Affiliations:** 1 Adjunct Faculty, School of Pharmacy & Health Sciences, University of the Pacific, Stockton, CA USA; 2 Adjunct Faculty, School of Pharmacy & Health Sciences, The University of the Pacific, 123 Forest Ave, Pacific Grove, CA 93950 USA

## Abstract

The function of proteins depends on their structure. The structural integrity of proteins is dynamic and depends on interacting nearby neighboring moieties that influence their properties and induce folding and structural changes. The conformational changes induced by these nearby neighbors in the micro-environmental milieu at that moment are guided by chemical or electrical bonding attractions.

There are few literature references that describe the potential for environmental milieu changes to disfavor SARS-CoV-2 attachment to a receptor for survival outside of a host. There are many studies on the effects of pH (acid and base balance) supporting its importance for protein structure and function, but few focus on pH role in extracellular or intracellular protein or actionable requirements of Covera-19.

‘Fold ‘em or Hold ‘em’ is seen by the various functions and effects of furin as it seeks an acidic milieu for action or compatible amino acid sequences which is currently aided by its histidine component and the structural changes of proteins as they enter or exit the host. Questions throughout the text are posed to focus on current thoughts as reviewing applicable COVID-19 translational research science in order to understand the complexities of Covid-19.

The pH needs of COVID-19 players and its journey through the human host and environment as well as some efficacious readily available repurposed drugs and out-of-the box and easily available treatments are reviewed.

## Introduction

Interest is redirected to the receptor surroundings and intracellular organelle environment by focus on furin and pH as two basic pillars of this translational thought experiment to demonstrate the importance for understanding some of the important science underlying COVID-19’s journey in humans. Using the current viral pandemic as a life saving model for therapeutic intervention by vaccines only and not other than ‘vaccine’ methods places ideational restrictions and impedes new treatment potentials. Several other treatment modalities for COVID-19 are introduced and supported by elegant studies and proven science. Viral activity, surface glycoproteins and interacting partners are affected by pH changes. Heat of ~104F [1] and washing/wiping surfaces with liquids/water and at a basic [alkaline] pH are important as are removable and replaceable paper or plastic covers on surfaces changed after every use.

The literature has exploded with hundreds of studies and reports on the various symptoms, demographics, outcomes and massive numbers of new facts regarding the current novel human pathologic agent SARS-CoV-2. The names 2019-nCoV or SARS-CoV-2 (the virus) and COVID-19, Covera-19 or Covera (the illness) are simplified herein to “CV19”.

The current appropriate priorities (washing, masking, social distancing, testing, tracking and isolation) will reduce new infections, if strictly enforced, available or doable. The psychological societ al costs and results of population-wide fears and anxieties induced by unscientific actions at all levels are not addressed in this paper.

The complexities of CV19 illness in the context of prior comorbid conditions, the many locations of receptor sites and mutations, based on the early New York hospitalization experience [2], are demonstrated and discussed using the early New York City model described below.

A study of the presenting symptoms of CV19 from 5700 hospitalized patients in the New York City area from March 1, 2020 to April 1, 2020 revealed ~ 60% males and ~ 40% females. The most common comorbidities were hypertension [~ 57%], obesity [~ 42%], and diabetes [~ 34%]. The presenting symptoms were fever [~ 31%] and a respiratory rate greater than 24 breaths per minute in ~ 17%, with ~ 28% of those receiving supplemental oxygen. Co-morbidities include cancer, cardiovascular disease [arrhythmias, ischemia, coronary artery disease (CAD), and congestive heart failure (CHF)], hypertension, asthma, COPD/emphysema, obstructive sleep apnea, obesity and older age. Few patients [6.1%] had no comorbidities immune suppression, aids/HIV, history of organ transplant, renal disease, liver disease, smoking (~ 16%). All patients tested positive by the second test for CV19. Of the patients admitted to the intensive care unit (ICU) for intensive care and ventilation support [~ 20% of 5700, or 1151 ICU admissions], ~ 25% or 282 ventilated patients died [2]. The requirement for ventilation represents ~ 2.28% of the total studied patient population. The mortality rate may continue to decrease as treatment improves and the number of tested asymptomatic CV19-negative or asymptomatic CV19-positive patients increases, assuming that the whole population is tested. Testing and measuring ‘hotspots’ measures ‘hotspots’ only and not whole population numbers may not be statistically valid, and are easily manipulated. The CV19 morbidity and mortality numbers cannot be accurate until the whole population is tested regardless of symptoms so a true population basis can be established for true statistical population comparison.

CV19 continues to prove that it is a potential serious threat to many populations, with some requiring rapid intervention and comprehensive care. Children are not as ‘immune’ to CV19 infection as initially thought. A recent JAMA article reported the clinical characteristics of many children with a pediatric inflammatory syndrome temporarily associated with CV19 [3], now called multisystem inflammatory syndrome in children (MIS-C), which can affect the heart, lungs, kidneys, brain, skin and eyes [4]. Is there a prolonged post-infection survivor syndrome in some patients? The T-lymphocytes are active but may potentially not be as effective as needed? A pandemic phylogenetic analysis of 84 distinct CV19 genomes in New York revealed entry paths from the US, Europe and the New York area [5].

The mortality statistics exhibit an elevated rate since many deaths are counted as due to CV19 if they are connected in any way. Deaths counted as ‘due to CV19’ actually may occur due to overwhelming sepsis and/or pneumonia. In reality, ‘due to CV19’ is a contributing factor that can be listed on its own line on the death certificate beneath the potential actual cause of death. Ventilator-treated CV19 patients who die have profound hypoxemia and may die of a myocardial infarction from insufficient oxygen or perhaps an arrhythmia. The immediate cause of death is myocardial infarction due to hypoxemia due to CV19 contributory effects (pneumonia or inflammation etc). Other practices are built into various semantics, as death certificate signers decide on death data certification. This is also found in other infectious diseases, such as influenza or HIV for example, which also have pneumonia and inflammation as co-morbidities, but the cause of death is listed commonly as Influenza and HIV/AIDS. Obviously this common practice is not strictly accurate, but is the current practice. Support for this reality has finally arrived from the National Center for Health Statistics, which states that only 6% of CV19-specific deaths are truthfully countable [6, 7]. Death ‘from’ CV19 is different from death ‘with’ or ‘due to (addition of)’ CV19. These separate lines are self-explanatory on the death certificate.

CV19 must accomplish the following in order to sustain itself and to multiply: Find a host > attach to and bind its receptor with the S1 spike protein > enter and travel through the receptor > initiate cell membrane fusion > create a pore and pass through to inside the host cell > enter an endosome > shed and leave the endosome to find the Host DNA and begin and finish the replication cycle > re-enter an exosome and travel to the host membranes > and ‘exit’ the host cell. The exit and leaving the exosome to ‘exit’ are not well established. Each of these steps has its own requirements and complexities including energy sources.

The mouth/oropharynx and nose as entrances for CV19-laden air and ‘droplets’ provide a direct path to the oropharynx, trachea, and lungs but also provide receptor sites along the way [8–10]. Two important angiotensin receptor blocker (ARB) proteins (ACE2[angiotensin converting enzyme 2] and TMPRSS2 [transmembrane protease serine 2]) are strongly expressed in nasal passage goblet secretory cells, type II pneumocytes of the alveoli in the lungs, and absorptive enterocytes of the intestine-ileum and lower bowel [9, 10].

Masking and distancing are important in personal prevention of both oral and nasal CV19 entry by potential aerosol and ‘droplet’ contagion. Masking does double duty—it prevents spread from infected patients with or without symptoms via CV19 aerosols or ‘droplets’ into the nasal passages and mouths/oropharynx of uninfected victims, and protects uninfected patients wearing masks. Surface contamination sources are a separate problem.

Ziegler et al. [9] noted other entry points and receptor site locations for CV19: the conjunctivae of the eyes, the epidermal surfaces of blood vessels, and the tissue targets of viral spread via blood vessels to target organs. Is an open wound or absent skin [i.e., burns] an opening for CV19? Burns and wounds expose blood vessels with receptor sites. The target organs include the eyes, lungs, heart, all types of blood vessels, brain (neural cortex and brain stem, spinal fluid), nose, liver, kidneys and intestines (ileal sites or lower bowel) [10].

The conjunctival entry point in the eyes presents special problems in covering and protection—perhaps wearing glasses may help, but that is questionable. In later CV19 illnesses, this entry may lead to conjunctivitis and be potentially restricted by tear production in response to irritation from CV19 presence. The conjunctivae also have goblet cells with a potential role in CV19 systemic spread. What is the role of tears or ocular fluids in combatting CV19 entry? Are the epidermal surfaces of blood vessels in the conjunctivae facilitating systemic CV19 entry? The pH of the conjunctival goblet cells and tears/ocular fluids was measured at pH 6.3/6.5 to 7.23/7.6 [11, 12], which may be advantageous to CV19 and its preference for acidic site activity. It is reported that borate may be a pH buffer in the conjunctivae and ocular fluids [11]. The conjunctivae as entry sites have been overshadowed by the emphasis on oral and nasal entry portals, hence the conjunctival CV19 entry site has received less attention and study. Loss of eyesight or ocular damage has not been mentioned in the CV19 literature. Does the higher pH range in the conjunctivae denature CV19 ‘S’ protein so it cannot be cleaved by furin for spike S1 subunit-receptor attachment? What is the furin level in the conjunctival goblet cells compared to oral and nasal passage sites? The answer may lie in the environmental milieu of the conjunctivae, which is bathed by tears and other ocular fluids. The conjunctival CV19 entry portal may support using a plastic facial mask that also covers the eyes as a superior infection barrier. The facial plastic mask may also limit PPE exposure from CV19 positive patients.

The gastrointestinal tract may be a ‘pass-through’ site for shed viral shells and proteins [10], but can also be an entry site from any source or from self-infection by asymptomatic positive individuals. The widespread systemic ACE2 and ARB (angiotensin converting enzyme 2 and angiotensin 2 receptor blocker sites) receptor distribution and viral entry may lead to multi-organ illness or potential multi-organ collapse and death.

## CV19’s intracellular journey and actions

Protein structure dictates function. Local environmental chemically or electrically induced conformational and structural changes [denaturation] may enhance or prevent agonist and host receptor proteins from interacting. Does ARB/ACE2 display a feedback loop in the CV19 entry process? After CV19 enters the cell via the ACE2/ARB receptor, uncoating of both CV19 and host membranes during the fusion process occurs, and the single-stranded RNA genome enters into the host cell cytoplasm via endosomal transport.

CV19 then initiates its reverse RNA reproduction cycle leading to eventual ‘exocytosis’, as its newly formed virions exit from inside newly formed exosomes to seek a new host cell’s ACE2/ARB receptors and repeat the process. The host is re-infected in a continuously expanding infection, both intracellularly and systemically. Are RNAs are made early from the first genes of the host DNA genome, and are multiple copies made? What is the fidelity of the subsequent mRNAs templated from the host DNA? Could these multiple copies be other sources of mutations if strict copying fidelity is not observed [13]? Where on the CV19 RNA and in what time-frame are the promoter and terminator genes active, if present at all? Where and when, early or late, on the CV19 RNA is this specific reverse transcriptase gene found? Is an enzyme involved in the production and initiation of this particular RNA reverse transcriptase? What will comparison with HIV reverse transcriptase models show? Could the built-in ‘degeneracy’ in the triplet genetic code (a different triplet code for the same amino acid in the mammalian genome) also be a potential adaptive evolutionary mechanism in CV19, as it uses only its RNA? It is generally accepted that, like poliovirus, when CV19 uses its reverse transcriptase to make its own RNA, protein synthesis ceases in the host. In support of this, Thoms et al. recently showed that “Nsp1 (nonstructural protein 1) from SARS-CoV-2 binds to the 40S ribosomal subunit, resulting in shutdown of host messenger RNA (mRNA) translation both in vitro and in cells.” [14]. Ban supports this by suggesting that Nsp1 suppresses host innate immune functions and interferes with mRNA binding, as the C-terminal domain of SARS-CoV-2 Nsp1 binds to the mRNA entry channel [15]. The high infection and transmissibility rate of CV19 was found to be due to another host protein called ‘neuropilin-1’, “which is recognized and bound by CV19’s spike protein and also facilitates CV19 cell entry and infectivity [16].” This finding seeks urgent study and comparison with newer emerging variants or other mutant entry proteins.

Additional intermediate conformational structures of proteins were addressed earlier by Bai and Englander [17], who stated that **“**All possible protein folding intermediates exist in equilibrium with the native protein at naïve as well as non-naïve conditions, with occupation determined by their free energy level.” Principles of protein structure and folding are illustrated by Walls [18], Wrobel [19], and Wrapp [20].

**PROTEIN GAME PLAYERS:** ‘Hold ‘em or fold ‘em or fold ‘em and hold ‘em?’

Wrapp et al. [20], Watanabe et al. [21] and Cai et al. [22] report that the ‘Spike (‘S’)’ glycoprotein mediates cell entry and cell fusion. The ‘S’ protein is described as a “trimeric class 1 fusion protein composed of two subunits: one responsible for receptor binding (S1 subunit) and a membrane fusion subunit on the S2 subunit. The ‘S’ protein undergoes a ‘hinge-like’ conformational change to expose the receptor-binding domain. The spike surface is dominated by host-derived glycans, with each trimer displaying 66 N-linked glycosylation sites” [21]. The ‘S1’ spike subunit binds to an amino terminal, and the ‘S2’ spike subunit binds to a carboxyl site after cleavage by furin [23]. Importantly, furin cleaves the ‘S’ protein of CV19 into two proteins: S1 and S2. As above, S1 is responsible for receptor site attachment, and S2, with its fusion peptide site, is responsible for fusion of the CV19 and host cell membranes to allow the CV19 genome to enter the host cell [22]. These actions may also indicate potential intrareceptor site mechanisms as CV19 transits through the receptor site. All of the receptor interactions and intracellular activities require an energy source. Once the membranes of both the host and CV19 are fused, the S2 fusion peptide forms a ‘fusion pore’ allowing the CV19 genome to enter the host cell for endosomal ‘entry’ and transportation [24].

Another potential CV19 entry-enabling protein was recently identified by Liu et al. [25]: heparan sulfate (SO4), which is closely related to heparin. Liu et al. also demonstrated that heparan SO4 removal inhibits CV19 attachment to the ACE2/ARB receptor site. Heparan SO4 is also known to be and described as an ‘adhesin’. Heparan SO4 provides an attachment site for CV19, plausibly holding it in the host receptor site area, and CV19 may not attach to the ACE2/ARB receptor site until it has adhered to the heparan SO4. Heparan SO4 attachment may be an early time-dependent or controlled event, and in this role it may enhance ‘adhesion’ of CV19 to the ARB/ACE2 receptor for subsequent actions to proceed. This ‘adherence and delivery’ action by heparan SO4 could be a critical early step in CV19 attachment to host cells because it ‘guarantees’ delivery of CV19 to the host cell receptor. Could heparan SO4 be blocked as a treatment, and the process halted at this early stage as well with prolonging time adherent dependency? Does heparanSO4 participate in a sequential process with other components? Heparan SO4 is a negatively charged polysaccharide that resided on the cell membrane. In the CV19 events sequence, it is derived from the host and onto the CV19 exterior and is ‘held’ in the host receptor site region. As Liu described removal or inactivation of heparan SO4 inhibits CV19 attachment to the ACE2/ARB receptor site [25]. Since heparan SO4 is a very acidic moiety, could it be inactivated by creating a basic environment and receptor site environment by pH adjustment? This may disallow an early receptor site CV19 attachment and CV19 cell entry? Hence creating an external basic environment may be an early potential CV19 entry denial into the host cell. This proposed model requires study and confirmation. CV19 is also bound by heparin, which leads to the question ‘does it uses heparin as a transport vehicle for systemic spread and infection, as HIV does with its platelet ‘taxi’ [26]‘? Use of heparin as a transport medium might help to explain the common coagulation problems of patients with CV19 infection because of heparin’s potential unavailability for normal blood anticoagulation and the widespread CV19 distribution. Yu et al. showed activation of an alternative complement pathway that blocks CV19 spike proteins [27]. Bouhaddou et al. [28] discussed the complicated phosphorylation processes during CV19 invasion. The increase in CV19 proteins and decrease in host proteins during various phases of phosphorylation in the energy production cycle showed that this decrease in host protein caused the demise of the affected host cell by inhibiting host mRNA translation and mitotic kinases [28].

## Furin

“Furin is a pro-protein convertase that cleaves the protein and amino acid chain region called ‘RXR/K/XR’ of precursor proteins and transforms the pro-proteins into biologically active proteins and peptides” [29]. Furin participates in many nuclear, intracellular, membrane and endosomal actions. Furin has important roles as a proteolytic cleaver of capsular polyprotein precursors prior to viral RNA assembly of newly made CV19 in the acidic environment of the trans-Golgi network.

Furin is a multi-functionally important protease that ‘senses’ its needed acidic pH environment necessary for function. The structure of furin has both an amino group and a carboxylic acid group but a net neutral charge, which plausibly allows ‘zwitterionic’ or dipolar-like behavior and functions. It is known that for furin action in various organelle sites requires an acidic pH [30]. The TMPRSS2 serine host protease in the receptor site of CV19 is acidic and hence a welcome partner for furin action. How ‘acidic’ or how ‘weakly basic’ a furin action site must be for function is not known. Other viral models may also provide acidic environments for furin activity and may be models for study and potential understanding of CV19. Furin is a common denominator in many cellular functions and acts as a ubiquitous cellular protein ‘concertmaster’. Could furin be artificially modified and targeted to achieve desired outcomes?

Pelleccia’s laboratory [31] identified furin as a companion protease to TMPRSS-2 in enhancing CV19 transit to the ACE2/ARB receptor for host cell entry. Furin affects spike glycoprotein structure cleavage in other viral evolutionary processes [19, 32] and has a long history as a protein ‘cleaver’. A four-amino-acid insertion between the edges of the S1 and S2 Spike protein subunits is ‘sensed’ by furin and allows furin-mediated ‘S’ protein cleavage [33]. Roebrook et al. found that furin requires a negatively charged four-amino-acid motif (or low-basic four-amino-acid sequence?) in the substrate-binding region to cleave that site [34]. This action enables the ‘open pre-attachment conformation’ necessary for the CV19 spike ‘S1’ protein subunit to attach to the ACE2 receptor site. What gene directs insertion of these four amino acids for recognition by furin cleavage, and can it be modified? The ubiquitous furin is present in the ARB/ACE2 receptor site before the ‘S’ protein arrives to be cleaved. The furin ‘S’ protein cleavage site was also described by Coutard et al., who noted its absence in other SARS-like coronaviruses (CoVs) [33]. The absence of the ‘S’ protein furin cleavage site in other SARS-like CoV viruses is an important difference. The presence of a four-amino-acid cleavage site attractive to furin could indicate CV19 mutations or an unusual evolutionary event. The S1 subunit has a basic N-terminus that binds to ACE2/ARB, while the S2 subunit has an acidic C-terminus that interacts with TMPRSS2 and furin after attachment for passage through the receptor site and contains the fusion peptide. These oppositely charged terminals may enhance furin’s ‘zwitterion’-like or ‘di-polar’ behavior and give it a greater capability to affect other protein sites. Given that CV19 mutates frequently for improved survival and function, any or all of the descriptions found to date and described here may change as CV19 mutates.

Cai et al. [22] and Roebrok et al. [34] support that the ‘S’ spike protein has both a ‘prefusion’ and a ‘postfusion’ state and that it undergoes the conformational change needed for successful host entry enabled by furin cleavage. They stated that the prefusion trimer structure has “three receptor-binding domains adjacent to the fusion peptide and that the postfusion structure has strategically placed N-linked glycans suggesting ‘viral protection’ against host immune responses and harsh external conditions” [22]. Wrobel et al. supported these observations [19]: “the human CV19 pathogen presents a more stable pre-cleavage form and an approximate 1,000-fold tighter binding of SARS-CoV-2 to human receptor than in bats.” They further state that “ these observations suggest that cleavage at the furin-cleavage site decreases the overall stability of SARS-CoV-2 and [but] facilitates the adoption of the open conformation change that is required for ‘S1’ to bind to the ACE2 receptor.”

Furin is implicated in mutations, tumor growth, viral and bacterial infections, protein cleavage and change into biologically active moieties. Some approaches have been suggested to stop furin activity. A furin inhibitor, a 2,5-dideoxystreptamine derivative, was designed to form a complex with furin [35] but needs further evaluation. Jean et al. [36] introduced alpha1-antitrypsin Portland as a ‘bioengineered’ serpin molecule selective for furin inhibition. They stated that the formation of a chemical moiety complexed with furin revealed activity inhibition of 100% complete after 2 min of exposure and described it as a “suicide substrate inhibitor’. Additional studies and clinical evaluations of furin inhibitors are expeditiously needed. Cheng et al. described several other furin inhibitors that may be useful after more supportive studies [37]. The use of furin blockers has not received enough attention given the critical function and presence of furin for most cell needs for protein cleavage, in the replication process and at the cell membrane receptor site. Other entities will likely continue to be identified concerning furin function as our knowledge and experience with CV19 or other viral agents grows [38]. Controlling and eradicating furin activity appears to be one of the primary keys to controlling CV19 and potentially other emerging viral pathogens. This action speaks to the fact that the singular target ‘vaccine’ focus has sidelined this and other treatment potentials.

A requirement for furin action, as for all cellular functions, is an energy source since biosystemic function is based on kinetic models instead of thermodynamic models.

## Mutation plays

Early in the CV19 outbreak, it became apparent that CV19 has the capability to evolve, as initially demonstrated by its movement from animal to human hosts [24]. This ability to mutate and adapt is still evolving.

The current intermediate protein forms of CV19 that emerge or mutate into different structures have increased potential for infection and improved host entry [25]. Intermediate forms may be found as new mutations emerge and thus give rise to a dynamic structural milieu. The ‘older’ forms will be replaced in time by the newer mutants. The ACE2/ARB receptor accommodates the current newly mutated form of CV19, which may signal a small or single.

CV19 has genome mutations at position 23,403, which are ‘adaptations’ to different geographical areas and their populations. This phenomenon was identified again recently [39] and indicates geographical mutations and changes toward increased infectivity. The ‘D614G’ (now named G614) mutation changes the virus spike protein that attaches to the CV19 receptor site. This effect partially demonstrates that an agonist can be modified for a better stoichiometric fit. Watanabe et al. [21] and Cai et al. [22] showed structural CV19 spike ‘S’ protein conformational changes but no receptor site changes.

The earliest mutation version, G614, found in the CV19 spike ‘S’ protein still ‘fits’ to the ACE2/ARB receptor protein but with stronger ability to fuse with host membranes and an at least 3–10 times greater infection capability, causing a mutation for improved survival but not a large structural change while maintaining attachment ability to the ACE2/ARB receptor protein, as confirmed by Choe and Fazan [26]. As mentioned above, G614 seeks comparison with the current ‘new’ variant to confirm its true appearance timeline. Infectivity appears to peak before symptom onset at ~ 4 to 7 days after exposure [23]. He et al. [40] estimated that 44% of infected individuals were infected during the ‘pre-symptomatic’ (asymptomatic) stage within household clusters and settings. These infections are primarily from close contact, absent masking and via aerosols, ‘droplets’ or surfaces.

The compilation of prior studies by Korber [39] also identified the G614 mutation and supports the earlier mutation model by Choe and Fazan [26], which shows that the ‘G614’ mutation is almost completely dominant in most countries and potentially now is being ‘re-discovered’. They also noted that ‘G614’ has “slightly changed the spike shape and protein structure to enhance and ease the viral membrane and host cell membrane fusion, which also supports a potential stronger infection rate of 3 to 10 times as before and that CV19 accumulates about two changes a month in its genome [26]“. These data may all be or become ‘moving targets’. In a larger sense, together with asymptomatic patients, non-masking and non-social distancing these mutations offer an explanation for the early and current rapid infection wave spreading across the globe, with a stronger affinity and infectious potential. The mutational ability of CV19 was further addressed and found to be more complex by Berrio et al., who importantly noted genome mutations independent of protein function impact [41].

Van Dorp et al’s report from the University College of London characterized “patterns of diversity of the SARS-CoV-2 virus genome” by identifying ~ 198 recurrent genetic mutations [42], which may help to explain how the virus adapts to its various environments, survival needs and human hosts by use of this large potential mutational repertoire. Are there epigenetic signals and activators for the mutations that may be identified? Regarding the known potential ~ 198 recurrent mutations [42], do they enable or guide CV19 and its ubiquitous player furin to a specific genome domain for mutational induction once inside the host environment in order to be more successful? It may help to think of this mutational induction as “functional genetic flow” enabled by ‘evolutionary pressure’ which may be guided by epigenetic need to adapt to changing requirements? The current mutations [or ‘variants’] may fit into the mutational potentials described above.

Baum et al. suggested developing an antibody cocktail to prevent CV19 from developing rapid mutational escape in response to the use of individual antibodies [43]. Mutational escape may make single target neutralizing antibody vaccines less effective. Hansen and Baum et al. reported on potential humanized neutralizing antibody cocktails for anti-SARS-CoV-2 use. This cocktail aims to decrease the potential, raised by Baum et al. [43], for the emergence of CV19 escape mutants from the use of single antibody vaccines [44]. The notion of single antibody vaccine failure due to a more rapid mutation potential is most concerning, and the above studies [42–44] must be solidly confirmed expeditiously and watched for close and appropriate response since this appears to be a looming event. The various cocktail antibodies work separately but synergistically. They [43, 44] also stated that this combination cocktail of survivor antibodies may overcome the presence of mutant forms that are present and have escaped treatment. Duffy questions why the CV19 mutation rates are so high [45]. The Medical Letter lists an early summary of CV19 therapy [46]. Konno et al. recently discovered a new interferon (IFN) antagonist which may positively impact therapy after confirmation and efficacy studies [47]. Impaired IFN responses are associated with CV19 disease.

What are the furin levels at this early stage in CV19 invasion? A comparison of entry genomes of the current mutations could indicate whether changed genomes are found as a ‘gene in situ tweak’ mutation to enable CV19. Could the pathologic process, once under way, induce other and more efficient mutations? Are there are more mutations early in the infection cycle, with fewer over time? Perhaps the ‘zwitterionic’ furin’s ability to ‘sense’ an acidic intracellular organelle site, adapt to its environment and maintain activity after mutation plays a role? Current CV19 genome testing is used as a method to determine the geographical spread of CV19. The papers by Watanabe et al. [21], Cai et al. [22] and Wrapp et al. [20] support the question ‘hold’ em or fold’ em’ and support that CV19 does both, depending on receptor site and intracellular actions, pH and CV19 mutational survival needs.

## Repurposed therapy and modifying the agonist and receptor

The pharmacology and biochemistry of new or repurposed drugs potentially efficacious in interfering with extra- and intracellular CV19 actions is a broad area. It is helpful to consider and select methods that disturb the flow of energy in the TCA/Krebs cycle and methods that could interfere with CV19 attempts at entry, membrane fusion for entry and exit, replication and protein reproduction. RNA mechanisms, furin cleavage, endo- and exosome activity at entry and exit are also important potential considerations for interfering with CV19. Every action and function within a cell requires a source of energy, and CV19 is no different.

The Medical Letter on Drugs and Therapeutics presents a large list of potential medications for CV19. Some potential therapeutic modalities will be discussed here. The Medical Letter does not address mutations and their therapy [46].

Earlier support of the protease TMPRSS-2 ‘activation’ of the CV19 Spike protein was offered by Glowacka et al. [48], and followed by a report from Kawase et al. [49] who used serine and cysteine protease inhibitors to prevent SARS-CoV entry [49]. Zhou et al. also suggested using protease inhibitors targeting CoV and select filovirus entry [50].

Hoffman et al. [51] then showed that the ‘S’ protein action is facilitated by TMPRSS-2, which is localized to the ACE2/ARB CV19 receptor domain. This activity also enables CV19 attachment and entrance to the cell via the ACE2/ARB receptor. Hoffman et al. [51] also reported that the ‘priming’ of spike agonist protein by TMPRSS-2 was inhibited by an FDA-approved orphan drug serine protease inhibitor, camostat mesylate, which is currently used for pancreatitis and esophagitis in Japan but could be available worldwide. Importantly, Hoffman et al. found that alveolar lung cells were not invaded by CV19 if camostat mesylate was used [51]. This is a critically important finding and therapy that has the potential to stop CV19 in its attacks on pulmonary tissues by blocking TMPRSS-2 ‘priming’ of the CV19 ‘S’ spike protein. Note that TMPRSS-2 has a serine as a part of its structure.

The FDA site on camostat mesylate describes its actions as follows [52]: “The mesylate salt form of camostat, an orally bioavailable, synthetic serine protease inhibitor, with (has) anti-inflammatory, antifibrotic, and potential antiviral activities. Upon oral administration, camostat and its metabolite 4-(4-guanidinebenzoyloxy) phenyl acetic acid (FOY 251) inhibit the activities of a variety of proteases, including trypsin, kallikrein, thrombin and plasmin, and C1r- and C1 esterases. Although the mechanism of action of camostat is not fully understood, trypsinogen activation in the pancreas is known to be a trigger in the development of pancreatitis. Camostat blocks the activation of trypsinogen to trypsin and the inflammatory cascade that follows. Camostat may also suppress the expression of the cytokines interleukin-1 (IL1b), Interleukin-6 [IL6] (known to be highly present in the lungs during CV19), tumor necrosis factor–alpha (TNF-a), and transforming growth factor-beta (TGF-beta), along with alpha-smooth muscle actin (alpha-SMA). These cytokines belong to the anti-inflammatory Th2 cellular immune response but are likely overwhelmed by the strong inflammatory opposing response. In addition, camostat inhibits the activity of TMPRSS2, the host cell serine protease that mediates viral cell entry for influenza virus and CoV, thereby inhibiting viral infection and follow-on replication [52]. Uno described camostat mesylate and its use, dosing and efficacy [53].

A current large-scale study of camostat is ongoing by the University of Aarhus in Denmark, with an endpoint in March 2021 [54].

Interleukin-6 [IL-6] blockade therapy has been used to reduce the macrophage inflammatory response, with some success [55]. As noted above, camostat may also block IL-6 and potentially support a stronger Th1-type cellular immune response.

The drug remdesivir produced a statistical improvement in nonspecific clinical status compared with that for standard care with ‘undefined clinical importance’ [56, 57] but has been approved for use. However, remdesivir may work better in combination with an IL-6 blocker or other antiviral drugs. Tortorici et al. suggested that strong human antibodies protect against CV19 [58], while Konno et al. reported on an interferon [IFN] antagonist [47]. Stauffer et al. mention using dexamethasone early [59].

Jurgeit et al. [60] report that the old anthelmintic drug niclosamide inhibits adenosine triphosphate (ATP) production by uncoupling oxidative phosphorylation and that it blocks endosomal acidification. Acidification of endosomes and acidic environments as discussed in this paper is a necessary environment for furin to be active in cellular endosomes, in the ARB receptor site, the fusion process of CV19 and host membranes, as well as Golgi body protein assembly. Niclosamide may become very useful once tried in human clinical scenarios. Niclosamide importantly also neutralizes endosomal and Golgi acidic environments [60]. Furin is a major actor in the acidic environments of the ARB receptor site, Golgi bodies and CV19 exit endosomal milieu. Further studies are expeditiously needed on niclosamide in the context of CV19 and other viral infections to support its potential CV19 and furin limiting actions by changing the environmental, intracellular, endosomal pH and TCA/Krebs cycle pathways. Niclosamide is available for repurposing and has been used safely as an anthelmintic for over 40 years in humans. Once shown to be dually useful as an oxidative phosphorylation inhibitor and organelle and endosomal pH changer in human targets or for CV19 therapy it may become a valuable re-purposed medication in treating CV19 or as a part of a medication cocktail as used in other illnesses. If found to do the same in human studies, then it can be considered safe for use since it has been used safely for over 40 years. It is plausibly desirable and useful for human CV19 or any current or potential emergent mutant or anti-viral therapy. Forty years of safe human use of niclosamide is sufficient to demonstrate recovery of its suppressive TCA/Krebs cycle activity and follow-on continued human health.

There are many repurposing drugs and identification efforts described in the literature, and it is beyond the scope of this paper cover them all. They may be useful adjunct therapy while awaiting vaccine roll-outs as back-up therapy, and efficacy expectations and results are studied.

Each activity in the cellular milieu requires a source of energy. This energy is derived from the ttricarboxylic acid cycle (Krebs cycle), as it shuttles various moieties through losses and additions of electrons and produces adenosine tri-phosphate (ATP), which eventually becomes cyclic adenosine monophosphate (cAMP) with serial loss of energy-laden phosphate (PO4) bonds at various sites in the TCA/Krebs cycle. cAMP then becomes the energy source for many cellular activities, including CV19 and furin actions. The relationships between phosphodiesterase (PDE) and cAMP are very complex and counterintuitive but deserve much more study for potential future understanding. If PDE is blocked, then cAMP is able to function. An increase in PDE could possibly prevent an energy source from enabling CV19 activity by blocking cAMP. Niclosamide [see above] also uncouples oxidative phosphorylation, and with its added endosomal acidic pH neutralization capability, as said, it could become a desirable drug in CV19 treatment. Aside from energy production interruption, furin also cannot act in acidic endosomal or other action sites without an energy source. Viral entry was also targeted as a strategy for broad-spectrum antivirals [61].

The well-described use of dexamethasone (6 mg daily) has shown promising results in combatting the inflammatory response in the lungs as an immune suppressive medication [59, 62]. This is a welcome short-term effect for inflammation suppression, but long-term Decadron or other corticosteroid use may suppress the immune system further toward potentially undesirable consequences. The use of different corticoid steroids showed an undefined lowering of 28-day mortality [63].

## Immune plays

There are currently many ongoing studies seeking an effective vaccine in clinical trials, with some declared efficacious, safe and being administered. Many studies and efforts have been gathered under the general term vaccine but are not a vaccine in the traditional sense. The word vaccine brings comfort. It is concerning that every solution to counter CV19 are single target efforts, and that CV19 in the face of efforts to destroy or incapacitate it may be able to change its mode of attack or molecular biology as it has the capability to mutate depending on geography and adversity. Other supporting concerns are production, distribution and cost to patients.

The effect of CV19 on the immune system appears to be a Th1-to-Th2 cellular immunity shift that allows infections, inflammation from macrophages and other problems to emerge. Th1 produces IFN gamma (IFN-G), increasing inflammation early, which is desirable in some cases of infection and illness, and Th2, whose cytokines include IL6, IL10, IP0 and macrophage inflammatory protein alpha, attract other Th2-acting reprogrammed macrophages and increases inflammation [64]. Via a very complicated pathway, monocyte-derived inflammatory macrophages add to the cytokine storm and blood coagulation pathway activation seen in late stages of CV19.

The cytokine Interleukin-6 [IL6] is also present in many cases, and IL6 blockade has been used with some success in many patients with macrophage-induced inflammatory syndrome [54]. The drug tocilizumab has been found to reduce the mortality risk of some ventilator patients by 45%, allowing them to be extubated or out of the hospital within a month. The researchers cautioned that dexamethasone therapy is undesirable at the same time with tocilizumab [65]. Tocilizumab is also reported to reduce cytokine release syndrome and IL6 action, reducing mortality [66, 67].

The role of monocyte-derived macrophages requires additional evaluation, including regarding the timing of signaling actions in order to discover their multifaceted function. Macrophages may play different roles in different tissues and environments. An excellent description of the complex macrophage action in the inflammatory setting and cytokine storms is found in the papers by Merad and Martin [64] and Schulert and Grom [55]. Macrophage-influenced ‘immune dysfunction’ or ‘overreaction’ is also commonly seen in some severe trauma patients with total system and tissue collapse from cytokine over-reaction [68]. A pertinent and helpful discussion of macrophage activity was found in the paper by Rydzynski-Moderbacher [69]. They pose that the macrophage response is more important than the antibody response. Macrophages derived from bone marrow monocytes circulate until they ‘find’ an intruder to arm themselves against and assist B cells in making antibodies and killer T cells in seeking CV19-infected cells for elimination. Other killer T-cells may also be sourced from survivor blood and transfused. Naïve T cells in the bone marrow of infected patients are a reservoir of useful blood cells, including other naïve white blood cells. The T-cells in the bone marrow could be harvested, multiplied to billions in number and given to a patient autologously via any available access routes. This procedure restores a ‘naïve and robust’ immune system that would act as a new Th1 infection-fighting immune system [70]. There are at least four ways to achieve a ‘younger and newer’ naïve immune system: by birth and inheritance, from cord blood, by early withdrawal and storage for need or use later [71], and by collecting a naïve and robust immune system from the bone marrow. If not drawn from cord blood at birth or stored later in life when healthy [71] and no immune suppression present, then the bone marrow site with only the white blood cells and macrophages harvested(not the stem cells) is always available. The white blood cells drawn from the above sources would give one a healthier and younger immune system to face any immune-suppressive situation or need. The bone marrow is also a resuscitation or IV infusion site for medications. The bone marrow reservoir of life-saving naïve white blood cells or red blood cells is always ready and available for extraction and use for health and/or life-saving needs. Intraosseous access for fluid and blood products transfusions is a common standard of care if intravenous access cannot be established. This therapeutic modality was presented as a poster and short discussion at the 2019 American Association for Advancement of Science (AAAS) meeting [70]. Mentioned by Charron [71] earlier and then Griffin [70] is that each of us has a new and renewable immune system in our bone marrow. This is a mixture with stem cells and macrophages, and the macrophages and or the stem cells may be harvested as needed. An immune system readily available for use may be plausibly considered as an available personal ‘vaccine’.

Absent from many reports older patients to CV19, as a co-morbidity is the fact that the immune system is also less functional as the patient ages. A recent timely article poignantly suggested that an aging immune system may allow exacerbation of CV19 illness and symptoms despite already presenting other known comorbidities [72].

Life-threatening illnesses, including pandemics, require investigating out-of-the-box and courageous visionary solutions based on sound science and studies.

Viral infections stimulate production of various types of interferons [IFNs] that induce an antiviral state [73], and some successes using IFNs to treat CV19 infections have been reported. Ziegler et al. [9] also reported the upregulation of ARB/ACE2 via an IFN-stimulated gene as a host viral infection defense. IFN alpha may induce response refractoriness, which requires close attention and possible changing or halting of IFN therapy.

IFN alpha has been used for over 50 years for treating HIV and hepatitis B and C safely. Grajales-Reyes and Colonna’s excellent discussion of IFN responses offers explanation and understanding of IFN roles in viral pneumonias [73]. The role of IFNs is complex and may be useful for CV19 treatment once completely studied, including safety, IFN responses and dose timing issues.

Studies of antibody signatures are emerging and relate to different outcomes. In a 22-patient cohort with the same Immunoglobin-G [IgG] levels, those who survived had spike-specific humoral antibody responses, while those who died had nucleocapsid-specific antibody elevations [74]. This finding was supported in an elegant study by Peng et al. [75], who reported that CV19-specific CD4+ and CD8+ T cell responses were found in most convalescent patients, while a significantly greater T- cell response was noted in those patients with severe illness. They concluded that “Differential subsets of CV19-specific T-cells can be associated with (specific) clinical outcomes.”

Matthew et al. reported that there are three immunotype profiles in CV19 patients: **1**. immunotype 1-associated disease severity with a “robust activated CD4 T-cell response, a paucity of circulating follicular helper cells (B cells? [GG]), activated CD8 T-cells, hyperactivated or exhausted CD8 cells and plasmablasts (PBs); **2.** immunotype 2 – characterized by less CD4+ cell activation, Tbet effector CD4 and CD8 T-cells, and proliferating memory B cells and not associated with disease severity; and **3.** immunotype 3-correlated negatively with disease severity and lacked obvious T and B cell responses.” [76]. A recent report indicates ‘ultrapotent’ antibodies identified from recovered patients’ sera that stop CV19 attachment to host cells while disrupting the infection machinery [58].

A systems biological assessment of immunity was presented by Arunachalam et al. [77], who noted that “The increase in pro-inflammatory mediators in the plasma, including IL6, TNFRNFS14, EN-RAGE, and OSM, coupled with suppressed innate immune responses in blood monocytes and dendritic cells (DCs) suggest a sepsis-like clinical condition.” In this context, it has been previously suggested that pro-inflammatory cytokines and bacterial products in the plasma may play pathogenic roles in sepsis, and the combination of these factors could be important in determining patient survival. Notably, the plasma of severe and ICU patients had significantly elevated levels of bacterial DNA, as measured by PCR quantitation of the bacterial 16S ribosomal RNA gene product. This finding was “correlated with bacterial DNA and the plasma levels of inflammatory mediators” [77]. The well-known infection and inflammation theory with danger-associated molecular patterns (DAMPs) and pathogen-associated molecular patterns (PAMPs) is supported by these findings. This finding [77] opens pathways for additional therapeutic methods and is a welcome step toward unravelling the complex inflammatory molecular actions in CV19.

CV19 patients with cytokine storms have fewer memory B cells, which are needed to develop a durable immune response. There appears to be a TNF alpha-mediated feedback loop that seems to shut down the germinal centers that produce memory B cells for the long-term anti-CV19 response [78]. Some immune responses are present but not as robust as those with B cells from germinal centers. The immune response to CV19 may be similar but not identical to the influenza immune response.

The IFN-G action and role bear repeating as IFN-G is related to the effect of catecholamines, specifically adrenergics. In a highly charged anxiety situation, a CV19 critically ill patient on a ventilator who has adrenergic stimulation (i.e., adrenalin, epinephrine) blocks the action of IFN-G by inducing a shift to a Th2 cellular response that is not beneficial at this point. The use of a beta blocker (B1 and B2 receptors, not B1 only) could restore the function of IFN-G, as was elegantly described by Prass et al. in brain injuries with ischemic stroke [79].

## The ‘droplet’ supremacy

A ‘droplet’ is a ‘droplet’, or is it something else?

It is generally accepted that CV19 travels from person to person on a droplet and that both are carried by the force of a sneeze or cough or some waft through air or as an aerosol. Webster defines ‘droplet’ as “a tiny drop, or a blob, driblet, drip, drop or a glob.” A ‘drop’ is further defined as “the quantity of fluid that falls in one spherical mass” and ‘a dose of medicine measured by drops or the smallest practical unit of liquid measure”. Wallensky further adds that the milieu includes an aerosol as a part of the ‘droplet cloud’, which is anything less than five microns in size, with droplets ranging from five to ten microns in size [80]. These definitions lead to a dilemma of defining what it is that CV19 actually travels on! Has anyone ever seen or proven that a CV19 is on within a ‘droplet’? Is there truly a structure that can be identified as the vehicle CV19 travels on/with or in? Can we modify the ‘droplet’ or a structure that CV19 travels on, with or amid so that CV 19 cannot escape, if it is indeed in, on or amid ‘droplets’? Is it truly carried ‘by’ or ‘with’ droplets or simply by the air flow from a cough, sneeze, breath or ‘by the wind’ as an aerosol or in ‘droplets? ‘Amid’ droplets may be an appropriate description since CV19 is likely amid a blob or a glob of moisture and other ‘stuff’ in the breath/wind or sputum of the CV19 carrier.

A recent paper addressed the issue of detection and quantification of CV19 by ‘droplet’ digital PCR from nasal swabs but failed to differentiate the actual ‘droplet’ status of CV19. However, additional useful data derived from the study could be used as a better and improved CV19 positive or negative test [81]. The pH of the ‘droplet’ cloud was not studied, and it may be useful to do so. At the ‘droplet’ stage of its accidental journey and search for an acceptable environment and receptor site, CV19 likely does not mutate. If the pH can be brought to an unsurvivable value for CV19 in the ‘droplet’ or air cloud or aerosol stage (i.e., to basic pH), then that may plausibly become a critically important timing and opportunistic strategy to stop host receptor site entry. All fluids and viral component proteins or amino acids within the ‘air’ also have an optimal and functional pH. Can the ‘air’ carrying CV19 be made slightly alkaline to decrease CV19 viability? Perhaps the masks worn by all can be impregnated by a basic environment-inducing agent to help incapacitate CV19 on contact? Studies of how long a CV19 particle ‘floats’ around in the ‘air’ are difficult to find.

## Environmental microenvironment management

CV19’s ability to enter the host cell is a purely accidental event. The stoichiometric matching structures and the acidic pH of receptor proteins are not accidental, but CV19 has a better hand to play than the potential host! How or if they affect each other stoichiometrically while attaching and passing through will dictate whether the proteins involved will hold ‘em or fold ‘em.

It has been reported that environmental UV-C light is effective for killing other viruses and kills CV19 on N95 masks [82]. If UV-C light kills CV19 on mask surfaces, why not on other surfaces? Far UV-C lights are already in use in hospitals to sterilize surgical supplies and instruments. The UV frequencies used (~ 200-225 nm) are safe and cause no damage. These UV lights could be easily installed in restaurants, buses, schools, cafeterias, theaters, and outdoors, as portable heaters are now in outdoor diners; the uses and potential small sterile areas are endless. The efficacy of UV-C lights depends on a simple distance vs effect relationship, and may not be a practical solution for CV19 eradication. It seeks expeditious study, experimentation and. This simple tool may offer a safe, useful and inexpensive expeditious solution to manage a part of the CV19 pandemic problem when coupled with surface covering, washing and potential eye protection.

Potential effects of radiation/ultrasound or other signal energy sources could be theoretically useful if the globular CV19 structures are all the same and if the spikes are all symmetrically positioned on each virus with predictable signal-dampening effects or potential resonance effects. There are studies of the CV19 viral coat structure, but none have evaluated simple spike location, separation or symmetricality. This information may be useful for eventual studies of outer CV19 coat destruction, disrupting its structural strength, integrity, and stability, and potential theoretical response to ultrasound or other energy-based resonance matched signal sources. UV light at resonant frequencies of ~ 222 nm seems to be efficacious in killing CV19 [82] on small areas and surfaces. Why is this particular frequency effective? Are there other frequencies that may be helpful to apply as above in order to kill CV19? How does this UV light and frequency kill CV19? Hand held UV-lights are used to clean surfaces and kill viruses and surgical supplies.

The proteins (‘S’, TMPRSS2, and furin) and their constituent amino acids or appendages in the CV19 spike and ACE2/ARB receptor are also prime study targets to determine their properties and potential for nonnative denaturation, environmental manipulation and pH changes.

The mouth/oral cavity and pharynx along with nasal passages or conjunctivae are the main entry portals for droplets and aerosols and can likely tolerate a larger change in pH to make the environment unfavorable for CV19. The nasal passages and oral cavity appear important as environmental modification targets because of the ACE2/ARB receptor sites found there. Is CV19 in the mouth/oropharynx still in, on or with its droplet on the way to the trachea and lungs? Are nasal passage studies are underway to investigate prevention of CV19 entry into nasal goblet cells using nasal sprays, nanobodies and old repurposed disinfecting solution applications [83]? Does CV19 travel to other organs via blood vessel distribution or by catching a ride on a protein ‘taxi’, such as heparin? For example, HIV hitches a ride on platelets [26]. There may be many viral properties similar to those of HIV or other viruses. External pH of CV19 environment as found in the host before or during acceptor site attachment and transit may be changed to an unacceptable pH for CV19 survival, within host tolerability.

## pH

The vascular receptor environment and most other pH-dependent sites for physiological and drug effects cannot tolerate a large swing in pH, with the exception of the duodenum, with a large pH swing of ~ 5.6–8.0. The normal blood/serum and overall tissue/body pH is ~ 7.35–7.45, which is tightly controlled by respiratory, renal and chemical/phosphate buffering systems. Furthe**r** studies may demonstrate a more general method of modifying the conformational milieu surrounding the receptor site by changing the local environmental pH within safe host physiologic parameters to induce denaturation. pH is defined as “a value to express acidity and alkalinity” based on the concentration of H+ ions by means of a logarithmic function defined by S.P.L Sorensen in 1909 as he wrote in his book “Ueber die Messung und die Bedeutung der Wasserstoff ionen konzentration bei enzymatischen Prozessen.” This may be translated into English as “On the measurement and the meaning of the acid ion concentration in enzymatic processes.”[Springer Verlag, 1909] It is important to understand that when the pH of a solution is decreased by one unit from neutral 7.0 to 6.9, the H+ concentration has increased tenfold. Small pH changes have tremendous consequences on protein structures and functions. Many current CV19 studies overlook environmental or pH effects on stoichiometric actions involving ligand or receptor site proteins or their intracellular, intranuclear and endosomal environments. While it is true that viruses do not have a pH per se, their glycoproteins and carbohydrate appendages do have optimal and minimal pH functional ranges. pH is of primary importance to proteins, receptors, and intracellular or nuclear micro-environmental functions. Thus pH manipulation may be a feasible method to affect protein structure changes, folding, denaturing and function. Serine, for example, as a part of the CV19 spike glycoprotein responsible for attachment to the ACE2/ARB receptor after being ‘activated’ by TMPRSS2 from the ACE2/ARB receptor, is acidic and could be amenable to a small environmental or pH manipulation toward the basic range and potentially inactivated as noted above in the heparan SO4 discussion. Both the ‘S’ protein, TMPRSS2 and furin have serine as a part of their structures. pH environmental changes cause protein ionization, which may induce morphological changes and an inability to function.

The body has many sites of varying pH that can accommodate receptor actions from many different drugs, chemical moieties, proteins, enzymes or microbes, including viruses. Denaturing by inhospitable pH causes protein biological and chemical activity to be lost or changed. All structural degrees beyond the primary structure are changed when a protein or nucleic acid is denatured. It is well known that extreme pH changes affect some peptide bonds, while serine and threonine are destroyed by an alkaline/basic environment. This phenomenon may provide an opportunity for study of a potentially important useful therapy. It is posited that environmental factors in the ACE2/ARB and ligand receptor surroundings affect protein structure and folding and that pH may be a likely factor. Protein folding or unfolding can be initiated by altering the pH. Will CV19 hold ‘em or fold ‘em in the event of a pH change sufficient to make a change in the structure and function, as described by Zhang [83]? It is highly plausible that a change to basic pH or less acidic pH may inhibit furin activity. Endosomal and Golgi acidity may also potentially be blocked with niclosamide, which is available on the US market as a repurposed drug.

As a rule, nothing is transported across a membrane in its ionized state. Changes in pH affect the amino acids and ionizable groups and residues of proteins. Once ionized by pH changes, folding of the protein can occur, dictating structural and functional changes [84]. Ionization and electrical properties are important in all stoichiometric receptor functions. In the case of CV19, the priming action of the ‘S’ protein changes it to an acceptable form by induced folding in a supportive pH acidic environment. It does not adopt its needed ‘induced fit’ until cleavage by furin. While we know that within the receptor site, the pH is acidic, we do not know any other features within the receptor site. For example, does the receptor channel have Na + or Ca++ gates that depend on other factors? What would be the effect of a pH change in and inside the receptor site region? The receptor site and the proteins of this active and mutating virus are likely sensitive to pH changes and manipulation, inducing denaturation. Denaturing [folding] a protein may include losing a hydrogen bond, a disulfide bond, a connector salt bridge, or a nonpolar covalent bond and cause loss of function. The need to tolerate small pH fluctuations to carry out their functions is generally characteristic of most cells. Talley and Alexov [85] address the issue of whether a protein or ‘action’ molecule can tolerate small alterations in pH and structure for function. They conclude by saying that “biological macromolecules have a necessity to tolerate small pH fluctuations”, “that such a scenario could be achieved if the pH-optimum of activity is similar to the characteristic pH of the subcellular compartment because the activity does not change much around the pH optimum”, and “at the same time, keeping activity unchanged upon pH fluctuations will require the 3D structure of the corresponding protein to maintain its structural integrity”.

The notion of variable and differential pH in different cellular components noted in this paper and its application to CV19 players is supported by Elferich et al. [86] as they explain pro-peptide ‘convertases’ and how they regulate activation of ‘their protease domains by sensing the organellar pH within the secretory pathway”. They support one of the basic notions posed in this paper by highlighting the relationship of furin and its relationship to organellar pH. Elferich et al. [86] related evolutionary analysis to the proportion of histidine residues within pro-peptide proteases, specifically furin. They state that furin “activates in the TGN (Tri-golgi network) at a pH of 6.5 (acidic).“ They reason that the acid-base balancing is slower than the histidine hydrogen exchange at basic pH values, noting the importance of the chemical reaction speed of different entities. They concluded by stating that “they demonstrated the pKa of the conserved histidine in pro-protein ‘convertase’ is acid shifted with furin and consistent with its lower pH of activation” [86]. An earlier paper by Feliciangeli et al. [87] identified a pH sensor in the furin pro-peptide that regulates enzyme activation and showed that furin activation occurs in the endoplasmic reticulum, and that furin has a conserved histidine that acts as a pH sensor. In support, Williamson et al. [30] further concluded that “Different spatial distributions of histidine residues modulate the activation pH of pro-protein convertases”, such as furin. Noted is also that furin may seek a specific amino acid sequence for cleavage supporting Coutard’s et al. report [33]. Talley and Alexov [85] state that “….only activity is biologically important, that macromolecules can tolerate small pH fluctuations that are inevitable with cellular function“, and that “our findings rationalize the efforts of correlating the pH of maximum stability and the characteristic pH of subcellular compartments since only the pH of activity is subject to evolutionary pressure”. More specifically, one of the central assertions of this paper is that the pH of the extracellular environment, receptor site and intracellular compartments appears critically important in enabling CV19.

Williamson et al. [88] reveal the pH-dependent activation of furin via a complicated combination of structural, mathematical and molecular dynamic simulations that suggest that “His-69 from the furin pro-domain serving as the pH sensor close to the TGN triggers movement of a loop region in the pro-peptide that modulates access to the cleavage site and thus allows for the tight pH regulation of furin activation”. Williamson et al’s [88] work establishes a model for further study, and potential furin control via environmental and pH manipulation, as espoused in this paper.

Acidic transmembrane glycoproteins have attached carbohydrate appendages whose response to minimal pH environmental changes may induce structural folding changes. One source states that the optimal pH for renal ACE2/ARB receptor activity is ~ 5.5–7.5 [89]. Does CV19 adapt to the various tissue environments via a small but still ACE2/ARB-compatible stoichiometric fit adjustment? The normal pH range in the oral cavity is large, ~ 6.8–7.5, from acidic to alkaline. The nasal cavity mucosa is 5.5–6.5 and increases to 7.2–8.3 with rhinitis. The fact that rhinitis caused by CV19 may also increase nasal pH has not been addressed and may also be a potential pH change CV19 therapeutic target since furin prefers an acidic environment for activity. Hull addressed the issue of changing nasal pH with nasal medicine application in patients afflicted with the common cold [90], with some success. It is plausible that CV19 may be destroyed or blocked by nasal receptor environmental pH adjustment [91]. England et al. found nasal pH to be a reliable parameter [92].

The notion of pH manipulation as a plausible therapy has been introduced for both acidic pre-entry, acidic intracellular Golgi body and endosomal pH manipulation [61]. It is apparent that furin favors an acidic environment for cleavage activity [86]. Manipulating the pH of select organelles and external environment appears a plausible method to stop furin which seeks acidic environments for activity. Potentially early heparanSO4 action could be interfered with a basic environment. An earlier report of pH dependent SARS coronavirus entry was mediated by the spike glycoprotein may have been a predictor of current entry events [93].

## Connecting thoughts: hold ‘em or fold ‘em?

The above discussions explore spike protein characteristics and chemistry, receptor site protein action, heparan SO4 & heparin involvement, pH effects on pertinent proteins, furin characteristics and activities, mutation effects, immune system action, select drug repurposing, energy needs and the role of PDE[phosphodiesterase], translational interpretations open to modification by newer data, an apparent Th1-to-Th2 cellular immune shift, potential use of far UV-C light, ‘droplet’ transmission, and niclosamide action on the TCA cycle and pH manipulation. The focus on environmental therapy and CV19 treatment is highlighted with some specific therapeutic recommendations.

Extra and intracellular environmental management may be possible even if and when the approaching agonist has the capability to mutate during current activity [as CV19 does] since each protein, including the mutated protein, has its optimal pH range, including and regardless of the current momentary structure. The optimum environmental factor modulating extracellular pH needed for protein function to proceed must be determined, sought and adjusted within the parameters of host and medication tolerance. Both extra or intracellular pH and host nuclear pH changes and their effect on furin await to be studied expeditiously at each step of CV19’s life cycle, from ‘droplets’ and air relationships to host entry, viral replication, exosome acquisition and exit from the host. Furin is a common denominator in many of these events. It must be learned: 1.How or if furin can be blocked or its functions modified by manipulating the pH, from its creation in the endoplasmic reticulum [87] or as it acts in organelles; 2. How to blunt furin pH sensing; 3. How to use furin blockers [94]; 4. How to use pH manipulation to stop CV19 and restore health.

Extracellular receptor proteins may be more easily influenced by environmental or pH adjustment since they are presumably more stable or easily affected proteins. An agonist/ligand may change/mutate and still ‘fit’ a nonmutated receptor.

The current batch of highly visible vaccines being developed has efficacy according to reports. Since CV19 has the potential to mutate on demand, will they still be effective, or is a new vaccine needed for each mutant? Environmental or pH adjustments for CV19 and ACE2/ARB protein changes have the potential to become a more universally effective therapeutic tool regardless of the current mutational structure or differing microbial or chemical agonists/ligands. As said the main steps in the infection pathway are random attachment, entry, fusion, replication, and endocytic and exocytic membrane site activity, with each step having its own requirements. There is still much to be learned about these steps, although enough may be known to begin clinical studies. This translational thought experiment is offered as a possible explanation and exploratory therapeutic beginning.

This translational thought experiment presents a potential coalescence of molecular environmental manipulation of pH in the context of ‘hold ‘em or fold ‘em’ and furin role interactions. Wu-Dunn and Spear support the environmental effect notions and focus in this paper [61] in an elegant and wide-ranging paper. Jurgeit et al. proposed the use of niclosamide beyond its current limited application [60]. As mentioned above, Niclosamide has been safely used for over 40 years in humans as an anthelmintic and has desirable and potentially efficacious actions in human illnesses and intracellular or intra-nuclear effects. Niclosamide uncouples oxidative phosphorylation and hence potentially denies a critical energy source for cellular, microbiological or viral and CV19 action [60, 61]. Niclosamide also blocks endosomal acidification. Could it also block receptor site acidification to potentially halt CV19 entry? These are known basic requirements for furin action, its production and activity [87]. Niclosamide is available as a drug in the US market and has widespread effects on viral infections that could be useful as one of several drugs in a cocktail (remdesivir, niclosamide, camostat mesylate, tocilizumab, other furin inhibitors or PDE manipulators, etc.) or potentially alone.

The discussed repurposed drugs meet the needs to stop CV19: halt host receptor site entry, interfere with the energy needs of CV19 to make proteins and exit (and the well-known increase in PDE inhibitory action on cAMP), as well as change the pH environment to further prevent furin sensing via histidine. The repurposed drug cocktail or combinations suggested in this paper and the cocktails suggested by Hansen [43] and Hansen and Baum [44] may allow the potential resuscitation of single-target vaccine failures due to CV19 potential in situ changes or adaptations and mutations. The predictions of multiple new mutations requiring new ‘vaccine’ development and multi-valent vaccine development herein are emerging now and seek expeditious attention lest we lose the progress made up to the present. The way forward, as are additions to ‘vaccines,’ is with multivalent vaccines combined with medicine or drug cocktails on CV19 as done with influenza and HIV therapies.

This translational thought experiment presents notions that speak to attachment action by proteins, functions of proteins (adhesion, attachment, receptor site function, fusion of cell membranes), protein production in acidic environments, cleavage by furin, sensing by furin, furin’s need for an acidic environment for action, insertion of specific and CV19-unique amino acid series for furin sensing in CV19, the role of histidine as a pH sensor, variability of histidine chemical reaction speed, proposed models of the roles of heparan-SO4, its removal and pH change for receptor site entry denial, mutations, and repurposed medications already available and shown to be safe. Various combination cocktails may be necessary for potential treatment of resistant mutations. Two major notions, furin control or destruction and the role pH management were advanced. Much of the uncertainty, the unknown nature and fear of CV19 could be ameliorated if we can control whether CV19 holds ‘em or folds ‘em. pH control may offer some predictability of CV19 action and behavior. It must be pointed out that a nationwide or global-wide vaccination campaign, with many requiring the scheduling of two separate vaccinations are labor and time intensive, and place a heavy burden upon most states, countries, patients, support labor forces as well as equipment needs and costs when compared to using re-purposed medications or medication cocktails. These may plausibly work as well upon study and trial, and are less labor intensive and costly. The nations unable to obtain or manufacture vaccines may be able to gain faster protection for their populations by using alternatives to vaccination upon validation of the alternative therapies and environmental notions offered here. While waiting for ‘vaccine’ availability issues to be solved the alternative potential solutions offered here may be a productive pathway while seeking and awaiting vaccines. The emerging mutations add urgency to seek alternatives to vaccines.

The current and future pandemics deserve all potential discovery and therapeutic ideas to be taken seriously and thoroughly evaluated, regardless of how ‘Swiftian’ they are or whether they have ever been considered before in this newer demanding context or not. Energy-dependent cellular activities potentially affecting host TCA/Krebs cycle functions will likely recover rapidly once CV19 is destroyed as they likely have for over 40 years with the use of niclosamide. To the above suggested cocktails, one can add a ‘newer and younger’ immune system from autologous white blood cell infusion by either prior storage or bone-marrow sources for potential expedited return to good health [70, 71]. Both reports reflect the essence and basis of translational thought experiments [70, 71].

The translational thought experiments posed and suggested herein may stimulate further ideation and study. Timely support for the hand that CV19 plays [Hold’em or Fold’em?] arrived from Goethe University in Frankfurt, where it was noted that ‘they have observed the RNA folding structures of the SARS-CoV-2 genome with which the virus controls the infection process” [95].

Enabling loop completion supporting reports include autophagy studies that point to endolysomal deacidification which may impede furin, [96], the association of autophagy with uncontrolled inflammation and delayed or absence of types I and III Interferons and increased cytokine production defects [97], and the plausible inhibition endosomal uncoating ‘thus preventing endosomal actions in entry and exit by CV19 (author’s comment)’. Autophagy control is thought to be via changes in activation phases of autophagy related genes [98]. These comments are merely superficial indicators of an extremely complex genetic interplay that is beginning to be unraveled.

Niclosamide is also confirmed by Gassen et al. [98] as a potential antiviral agent as suggested in this paper before it began its prolonged editorial and review journey. The role of cystidine peptidase control is thought to impede cell entry and replication [99]. Pislar et al. [99] note that cathepsin inhibitors ‘dual’ inhibitory action on viral and host by lessening the positive immune response may support the needed multi-pronged therapeutic approach as suggested earlier in this paper by Baum et al. [43] and Hansen et al. [44] who also suggest a multi-pronged therapeutic approach to counter mutation escape by viral variants,

In addressing the activity at and of the furin cleavage site, Xing et al. [100] note that many mutations are active at that site, and may lessen the importance of the cleavage site. Their interesting studies seek more supporting data to unravel a complex finding. Xia et al. [101] discuss the role of trypsin in the furin cleavage site and its mutation or change from a ‘RRAR’ configuration into a ‘SSAR’ configuration which appears equally effective in cleavage and viral entry, adding more confusion to a most complex and vexing situation.

Pardhan et al. [102] review and support that the most significant ocular symptoms by patients with CV19 infection are ‘sore eyes’. However, one source reports that there was loss of vision by a patient infected by CV19 [103].

## Conclusion

Translational thought experiments add value to science since without them science would not progress. Translational experimental ideation, based on and referenced with sound and proven science, deserves acknowledgment and recognition as a necessary equal partner in scientific or medical discovery and progress. To do so will encourage huge scientific progress and pave the way to the future.

Will the question between Darwin’s natural selection, where only those organisms best adapted to their environment survive, and Lamarck’s notion of adaptive force, where organisms can alter themselves to meet the needs of their environments be answered? The roles of furin and pH appear to be in the midst of the chaotic environment that allows CV19 to merge Darwin’s science with Lamarck’s contributions. Regardless of the pH of the moment, whether external, receptor site, internal cell site or organelle, pH becomes a major determinant of protein or chemical structure [folding] and function. If histidine sensing disappears or amino acid sequences change by mutation or evolution, CV19 and its minion furin can still be managed within host tolerance of pH management. Furin works best in an acidic environment. The newest emerging ‘mutant’ has an optimal pH for function, and population penetrance may be determined by pH influence on the players and their momentary managed environmental pH. CV19 will change and mutate, but the role and importance of pH remains unchanged. pH management allows CV19 to hold’em or to fold’em and plausibly grants loop completion of this translational thought experiment and potential global application of non-vaccine therapy. Both Darwin and Lamarck are correct and imply separate but equal historical scientific truths.

## Data Availability

Not Applicable.

## Abbreviations

SARS-CoV-2: Severe Acute Respiratory Syndrome-Covera-2
pH: Designates acid & base ratios
Covera-19, Covid-19: Refers to the current pandemic virus and often the disease
CV19: Refers to all three as above in this paper
CAD: Coronary artery disease
COPD: Chronic pulmonary obstructive disease or emphysema
CHF: Congestive heart failure
HIV: Human immunodeficiency virus
ICU: Intensive care unit
JAMA: Journal of the American Medical Association
MIS-C: Multi-system inflammatory syndrome in children
T-cell: Thymus derived immune cell of the cellular immune system
etc.: Etcetera
ARB: Angiotensin Receptor Blocker, hypertension medicine
ACE: Angiotensin Converting Enzyme, in this context is the receptor for CV19/SARS-CoV-2
TMPRSS-2: Transmembrane protease serine, one of the receptor site proteins of the host, aids in ‘priming’ the spike protein for receptor site entry.
RNA/mRNA: Ribonucleic acid/messenger ribonucleic acid = ‘m’ refers to messenger and that it copies the DNA of the host and transfers it to the RNA with a transfer RNA (tRNA)
DNA: Deoxyribonucleic acid is the basis of the genetic code for all living beings
Nsp1: Nonstructuralprotein1 is the protein that shuts down host protein and RNA processes after viral RNA copies host DNA
40s&16s: Refers to ‘Svedberg’ units, describing the rate of descent and gravitational pull on RNA units settling on the bottom of a flask and is a measure of RNA size
‘S,S1&S2’: Spike proteins. ‘S’ is the un-cleaved initial spike protein, and S1 and S2 are the subunits after furin cleavage that enables S1 receptor entry & passage, with S2 fusing CV19 & host membranes and making a pore for CV19 RNA genome entry via a created ‘pore’ for endosomal move to nucleus “RXR/K/XR” refers to a specific amino acid sequence that furin seeks out to cleave the ‘S’ protein into S1 & S2 spike protein subunits.
DAMPS & PAMPS: Damage or Pathogen Associated Molecular Pattern from a current well known theory of infection and disease caused by pathogens and/or microbes and cellular/tissue damage
UV-C: Ultraviolet light C with a certain frequency-ie ~ 220 nm
D614G, G614: Series of mutants of CV19 thought to be early identified mutants that had greater Infectivity of at least ten times as fast as before, and thought responsible for the rapid worldwide spread earlier
C1&C1r: ‘Complement and complex’ describing the activation of the antibody & antigen system
IL1b, IL6, IN gamma, IL10, TNF alfa: Series of cytokines active in cellular immunity responding to immune system needs, with IL6 being the important cytokine causing the inflammatory reaction that is potentially most dangerous from the pneumonia by CV19
alpha SMA: Alpha smooth muscle actin is the chemical most active in causin fibrin deposition in smooth muscle found in blood vessels and pulmonary tissues etc.
Th1&Th2: The two major branches of the cellular immune system; derived from CD4+ the Th1 ‘helper’ cells produce cytokines that help in fighting infection etc.; Th2 type cells are involved in autoimmune or anti-inflammatory/allergic responses
ATP, cAMP: Adenosine tri phosphate (with three high energy phosphate bonds), and cyclic adenosine monophosphate (after ATP donates energy from losing two phosphate bonds) is the main source of energy for the furin and RNA viral protein generation
TCA: Tricarboxylic acid cycle is another name for the Krebs cycle
PDE: Phosphodiesterase is the enzyme that controls cAMP energy release-by inhibiting PDE the levels of cAMP decrease and CV19 or furin are inhibited. cAMP is also called a’second messenger’
TNFRNFS14: Tumor necrosis factor apoptosis inducing moieties
EN-RAGE: Calcium binding protein that increases inflammation
OSM: Refers to osmolarity and diffusion
DCS: Refers to dendritic cells and antigen presenting action
PCR: Polymerase chain reaction that amplifies minute amounts of DNA, and is used to measure the presence of DNA of microbes, in this context of CV19 taken from a nasal swab, or other source
